# Thermal gradient ring for analysis of temperature-dependent behaviors involving TRP channels in mice

**DOI:** 10.1186/s12576-024-00903-w

**Published:** 2024-02-08

**Authors:** Tomoyo Ujisawa, Jing Lei, Makiko Kashio, Makoto Tominaga

**Affiliations:** 1 Division of Cell Signaling, National Institute for Physiological Sciences, National Institutes of Natural Sciences, Okazaki, Japan; 2 Thermal Biology Group, Exploratory Research Center on Life and Living Systems (ExCELLS), National Institutes of Natural Sciences, Okazaki, Japan; 3 Course of Physiological Sciences, The Graduate University for Advanced Studies (SOKENDAI), Okazaki, Japan

## Abstract

There are a lot of temperature-sensitive proteins including transient receptor potential (TRP) channels. Some TRP channels are temperature receptors having specific activation temperatures in vitro that are within the physiological temperature range. Mice deficient in specific TRP channels show abnormal thermal behaviors, but the role of TRP channels in these behaviors is not fully understood. The Thermal Gradient Ring is a new apparatus that allows mice to freely move around the ring floor and not stay in a corner. The system can analyze various factors (e.g., ‘Spent time’, ‘Travel distance’, ‘Moving speed’, ‘Acceleration’) associated with temperature-dependent behaviors of TRP-deficient mice. For example, the Ring system clearly discriminated differences in temperature-dependent phenotypes between mice with diabetic peripheral neuropathy and TRPV1^**−/−**^ mice, and demonstrated the importance of TRPV3 in temperature detection in skin. Studies using the Thermal Gradient Ring system can increase understanding of the molecular basis of thermal behaviors in mice and in turn help develop strategies to affect responses to different temperature conditions in humans.

## Temperature sensing in mammals

Animals have evolved sophisticated physiological systems for sensing ambient temperature since changes in environmental temperatures can affect various biological processes [1]. Somatosensory neurons are generally assumed to mediate sensing of external temperature by converting temperature information to neural activity via afferent input to the central nervous system. Upon relay of sensory information to the cerebral cortex through the thalamus, temperature information is perceived and discriminated [2]. However, this pathway is not associated with thermal behaviors. Instead, another pathway that connects the spinal cord to the lateral parabrachial nucleus (LPB) and preoptic area (POA) mediates generation of thermal behaviors, which arise before the thermal challenge impacts core body temperature [3]. This spinal-LPB-POA pathway also leads to autonomic heat-gain responses, such as shivering thermogenesis and cutaneous vasoconstriction. Warmth-activated dorsal LPB neurons transmit signals to the median POA (MnPO). These signals activate GABAergic projection neurons in the POA that in turn inhibit excitatory pathways that drive sympathetic thermoregulatory effectors, leading to reduced thermogenesis and cutaneous vasodilation. Conversely, cold-activated LPB neurons provide glutamatergic excitation to GABAergic interneurons (rather than to projection neurons) in the MnPO that reduces the activity of inhibitory projection neurons in POA neurons. This cold-activated pathway leads to attenuation of cutaneous vasodilation and an increase in heat production.

## Thermosensitive TRP channels

Somatosensory neurons consist of various populations of temperature-sensitive proteins, and the neurons have specialized gene expression of thermosensitive transient receptor potential (TRP) ion channels [4, 5]. Thermosensitive TRP channels are responsible for thermal transduction at the peripheral ends of somatosensory neurons and function over a wide range of temperatures. Activation of thermosensitive TRP channels causes cation influx, leading to membrane depolarization that activates voltage-gated Na^+^ channels and generates action potentials. This mechanism is a simple, but effective way to convert temperature stimulus into electro signals.

A prototypical TRP channel was identified in eyes of mutant *Drosophila* that exhibits transient, but not continuous, changes in receptor potentials in response to light stimulus [6]. Accordingly, TRP channel research is most advanced in *Drosophila*, but *Drosophila* TRP channel family members and functions differ substantially from those in mammals. In mammals, there are 28 TRP channels in 6 subfamilies: TRPC (canonical), TRPM (melastatin), TRPV (vanilloid), TRPML (mucolipin), TRPP (polycystin), and TRPA (ankyrin). Since the initial cloning of TRPV1 in 1997, there are now 11 thermosensitive TRP channels (TRPV1, TRPV2, TRPV3, TRPV4, TRPM2, TRPM3, TRPM4, TRPM5, TRPM8, TRPC5, and TRPA1), many of which belong to the TRPM and TRPV subfamilies [4, 5]. Although there are many thermosensitive TRP channels, only a few TRP channels have clearly demonstrated heat-gated activation. Structures for all 11 thermosensitive TRP channels were solved at an atomic level with cryo-EM [7–16], yet the mechanism by which temperature induces opening of these channels remains unclear.

Many reports showed that thermal behaviors in mice lacking thermosensitive TRP channels differ from those of wild-type (WT) mice. In a linear thermal gradient system or a two-plate test, TRPM8^−/−^ or TRPV3^−/−^ mice were reported to show temperature preferences for the cooler side compared to WT mice [17–19]. These mutant mice also stayed in the coldest zones (below 16 °C) for longer than WT mice. In another study, TRPV4^−/−^ mice preferred the warmer side [20]. At temperatures below 50 °C, TRPV1^−/−^ and WT mice had similar temperature preferences [21], even though the temperature thresholds for heat-evoked activation of TRPV1 are about 43 °C in vitro. However, in a hot plate test TRPV1^−/−^ mice showed less sensitivity to temperatures above 52 °C than did WT mice [22], and TRPM2^−/−^ mice did not recognize differences between 33 °C and 38 °C [23]. The temperature sensitivity of TRPA1 is still controversial [24, 25]. Mouse TRPA1 activation was initially reported for cold temperatures below 17 °C, which is in the noxious range [26]. However, two other studies involving TRPA1^−/−^ mice found no difference in [27] or reduced [28] cold sensitivity between WT and TRPA1^−/−^ mice. Furthermore, TRPA1, together with TRPV1 and TRPM3, was recently reported to be involved in sensing of acute noxious heat [29]. TRPV1/TRPA1/TRPM3 triple knockout mice did not respond to noxious heat stimuli, whereas responses persisted in single knockout mice. Expression of the cold-sensitive channel TRPM8 is required for warmth perception in mice [30]. Warmth perception in the mouse forepaw is regulated by C-fibers that are either activated or inhibited by warmth. TRPM8^−/−^ mice lack warmth-inhibited C-fibers and thus do not respond to a temperature increase from 32 °C to 42 °C, suggesting that signaling from warmth-activated C-fibers alone is not sufficient for warmth perception.

## Thermal gradient ring system

**Fig. 1:**
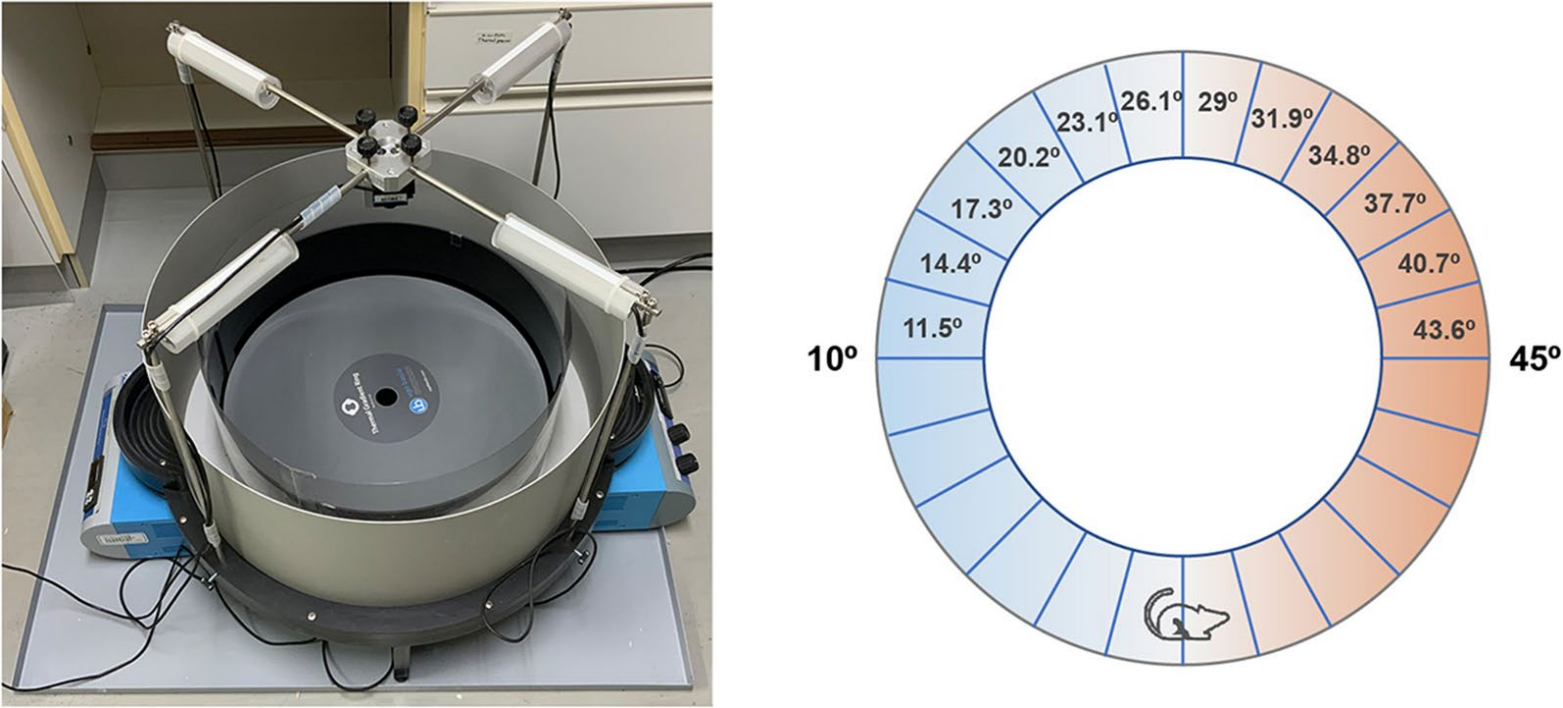
Thermal Gradient Ring system. The Thermal Gradient Ring viewed from above (left) and a schematic representation of the temperature gradient zones with cooler and warmer temperatures shown in blue and red, respectively (right). Two temperature control devices under the ring create a thermal gradient on the floor of the apparatus. There are 12 zones on one half of the ring. These zones and the gradient are duplicated on the other half

Previous studies examining thermal behaviors have several limitations [17, 18, 21]. For example, these studies used methods like a two-plate test that does not have high temperature resolution. Meanwhile, the linear thermal gradient method does not preclude the preference of mice to stay in a corner. A stereotypical mouse behavior is to hide in the corner, which can introduce a bias when mouse behaviors are analyzed with cold or hot zones positioned in corners. The Thermal Gradient Ring system was introduced to overcome these disadvantages[31]. The Thermal Gradient Ring (Fig. 1) can be less stressful to mice than the two-plate test, and the mice can move freely on the ring rather than remaining in a corner.

The Thermal Gradient Ring is 12 cm high with a 45 cm and 57 cm inner and outer diameter, respectively (35560-F, Ugo Basile SRL, Italy). An infrared camera (C920, Logicool, Switzerland) placed on the upper side of the apparatus allows monitoring of mouse behaviors in the dark. Part of the inner wall of the apparatus is transparent so that the camera has no blind spots. Cooling and warming devices in the apparatus allow analysis across a range of temperatures (e.g., 10 °C to 45 °C with a Δ2.9 °C temperature gradient). The apparatus is further equipped with a system that records the floor temperature in the middle of each zone such that at room temperature (25 °C), which is close to thermoneutral, the middle of the coldest or warmest zone is marked as 11.5 °C and 43.6 °C, respectively [32]. If necessary, different room temperature conditions can be used to evaluate how the floor temperature and room temperature affect mouse behaviors. The resolution of temperature differences can also be increased. For example, a range from 19 °C to 36 °C can be achieved by setting the coldest and warmest zone as 19.7 °C and 35.3 °C, respectively, with a Δ1.4 °C temperature gradient. To generate a noxious cold temperature condition, a temperature range from 6 °C to 49 °C with a temperature gradient of around Δ3.5 °C can be used. In this case, the middle of the coldest zone is 7.8 °C. Parameters such as ‘Spent time’, ‘Travel distance’, and ‘Moving speed’ are automatically analyzed using ANY-maze software (Stoelting Co.). ‘Moving speed’ is calculated every 0.1 s when the mouse moves and no calculation is made when the mouse does not move. These data can be used to calculate ‘Acceleration’ with the following equation: m/s^2^ = [v(a)−v(b)]/[t(a)-t(b)] where (b) refers to the time point before time point for (a) and v and t indicate speed and time position, respectively.

**Fig. 2:**
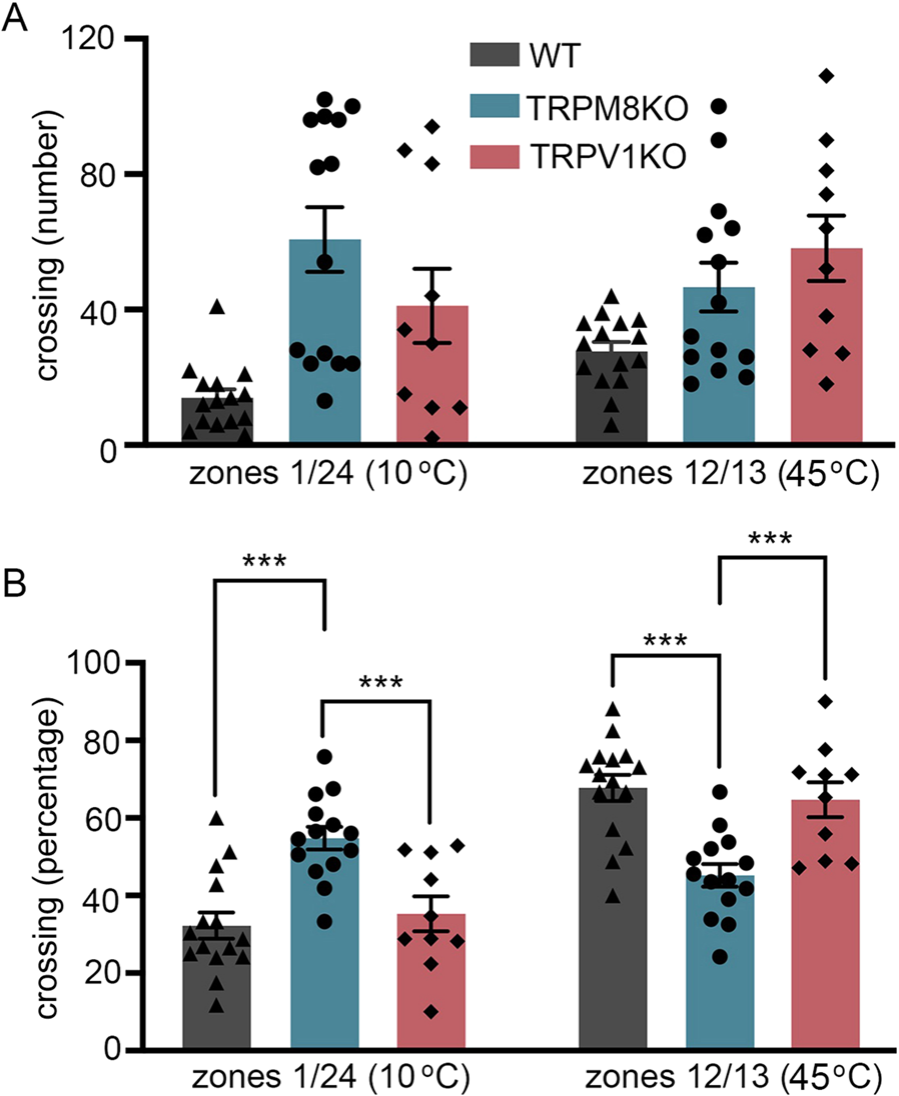
Analysis of crossing behaviors to warmer and cooler temperature zones. **A** Comparison of number of crossings from 1/24 zones (10 °C) or 12/13 zones (45 °C) in 60 min made by wild-type (WT, n = 15), TRPM8KO (n = 14) and TRPV1KO (n = 10) mice. **B** Comparison of percentage of mice that crossed 1/24 zones or 12/13 zones in 60 min among WT (n = 15), TRPM8KO (n = 14) and TRPV1KO (n = 10) mice. *** p < 0.001 by one-way ANOVA multiple comparison

Previous studies of thermal behaviors in mice focused mainly on ‘Spent time’ in each temperature zone. However, a focus on Spent time does not address several questions regarding relationships between temperature-sensitive proteins and thermal behaviors. In our study, measurement of multiple thermal behavior parameters, including ‘Travel distance’ and ‘Moving speed’ in a 60 min measurement period was usually sufficient to fully characterize temperature-dependent behaviors in mice. Based on the ‘Spent time’ result alone, determining at which temperature the mice felt comfortable was difficult, whereas inclusion of other parameters like ‘Moving speed’ allowed the temperature to be defined. From the mean of ‘Acceleration’ data, we found that all genotypes (WT and several TRPKO mice which we examined), except TRPM8^−/−^ mice, had an accelerated ‘Moving speed’ at temperatures below 14.4 °C, suggesting that these mice recognize noxious cold temperatures. Conversely, TRPM8^−/−^ mice showed no evasive behaviors in the low temperature zones [32]. In this regard, we newly analyzed previously obtained data to determine the number of crossings at zones 1/24 (10 °C) and zones 12/13 (45 °C) [32]. When mice moved to warmer zones (12/13), they can cross zones or turn back. Similarly, mice can cross zones (1/24) or turn back to move to the cooler zones. WT and TRPV1KO mice avoided crossing zones 1/24, while TRPM8KO mice did not (Fig. 2A). These results are consistent with the fact that TRPM8 is activated by cold temperatures. However, WT mice moved less compared with TRPV1KO or TRPM8KO mice as we reported before [32]. In a comparison of the percentage of mice that crossed zones (Fig. 2B), there was a statistically significant difference in which WT and TRPV1KO mice crossed high temperature zones (12/13) more frequently and TRPM8KO mice crossed low temperature zones (1/24) more frequently. Thus, we can perform various analyses concerning temperature-dependent behaviors that can be determined by the variance between preference and avoidance of different temperature zones.

Body temperature is measured by inserting a temperature logger (nano tag, ACOS, Nagano, Japan) in the mice, either in the intraperitoneal cavity or subcutaneously in the back [32]. The logger is controlled by FeliCa (RC-S380, SONY, Japan) to start or finish a measurement. Results are loaded using Felica and monitored using nano tag viewer software. Mice are nocturnal animals and move more at night, when they also have higher body temperatures. Circadian changes occur for both subcutaneous and core temperatures, which are high at night and lower during the day; subcutaneous temperatures are approximately 2 °C lower than core body temperatures. The mice had elevated subcutaneous and core temperatures after they were placed on the ring apparatus, likely because they began exploring. The temperatures then decreased over time. Both the subcutaneous and core temperatures similarly change during the daytime and nighttime, with high temperatures at nighttime indicating that initial body temperatures do not affect body temperature in the thermal behavior assay. As such, all experiments could be conducted during the day [32].

Based on these conditions, a new analysis using multiple parameters of the Thermal Gradient Ring to examine various TRP-deficient mice was proposed [32]. Each half of the ring has a duplicate temperature gradient. This design increases the accuracy of behavior results since mice can readily pass through any zone and have no opportunities to hide. WT mice showed no obvious temperature preferences in the first 20 min of the experiment, likely because the mice first explored their preferred temperatures. Eventually, the mice chose regions having specific temperatures where they remained for the last 20 min of the experiment.

## Monitoring temperature-dependent behaviors with the thermal gradient ring system

The Thermal Gradient Ring allows detailed characterization of temperature-dependent behaviors of WT mice and mice lacking various proteins including thermosensitive TRP channels [32]. Data obtained with the Thermal Gradient Ring are consistent with data obtained with other methods, but the Ring system has higher temperature resolution and better reflects the balance between preference and avoidance. In one previous report using a cold plate assay, TRPA1^−/−^ mice showed fewer paw lifts at 0 °C than WT mice [28], but another report showed no difference between WT and TRPA1^−/−^ mice [27]. In a Thermal Gradient Ring assay, TRPA1^−/−^ mice showed similar ‘Spent time’ in all zones as WT mice, but they more quickly recognized warmer zones as being preferable [31]. Another study using the Thermal Gradient Ring showed that only TRPA1^−/−^ mice had a preference for 33.9 °C as early as 20–40 min in a thermal gradient of 19.7 °C to 35.3 °C, while clear temperature preferences were exhibited for all genotypes with a 11.5 °C to 43.6 °C thermal gradient. TRPA1^−/−^ mice did not enter either cold (< 11.4 °C) or hot (54.9 °C) temperature zones, indicating that TRPA1^−/−^ mice avoided noxious temperatures. These results suggest that TRPA1^−/−^ mice have a high temperature sensitivity as suggested recently [29]. The mean of ‘Acceleration’ data, showed that all genotypes, except TRPM8^−/−^ mice, had an accelerated ‘Moving speed’ at temperatures < 14.4 °C, suggesting that these mice recognized noxious cold temperatures. Conversely, TRPM8^−/−^ mice showed no evasive behaviors in the low temperature zones [32].

All thermosensitive TRP channels have temperature thresholds for activation, as determined in vitro. However, defining relationships between the reported temperature thresholds and thermal behaviors is difficult. For example, the activation temperature thresholds for TRPV3 and TRPV4 are 32 °C to 39 °C and 27 °C to 35 °C, respectively. TRPV3^−/−^ mice showed significantly more ‘Spent time’ in the 23.1 °C zone, which is far from the temperature range at which TRPV3 is activated. TRPV4^−/−^ mice preferred to stay in zones from 29.0 °C to 31.9 °C, which is slightly cooler than the preference of WT mice [32]. TRPM2^−/−^ mice spent similar amounts of time in the 31.9 °C to 37.7 °C zones, whereas WT mice preferred the 34.8 °C zone over the 31.9 °C or 37.7 °C zones [32]. This result suggests that TRPM2^−/−^ mice do not recognize temperature differences between 31.9 °C and 37.7 °C, which is consistent with the results of a previous study [23]. TRPV1^−/−^ mice, but not WT mice, entered the 50.0 °C zone during the 40–60 min observation period with a thermal gradient of 14.6 °C to 54.9 °C, which corresponds to activation of TRPV1 by temperatures > 43 °C in vitro [33, 34].

## Analysis of neuropathic pain in mice using the thermal gradient ring

Thermal Gradient Ring not only enables the direct assessment of TRP-dependent thermal phenotypes, but also allows the evaluation of temperature hypersensitivity induced in mouse models of neuropathic pain[35]. Valek et al. observed that mice with a specific knockout of neuronal nucleoredoxin (oxidoreductase) displayed hyperalgesia to both cold and heat in the Thermal Gradient Ring, a phenomenon attributed to increased calcium through TRP channels[36]. Xue et al. investigated a chronic pain model carrying *Scn9a*^*R185H*^, a gene encoding the voltage-gated sodium channel Nav1.7. They found that the pathological mutation R185H, identified in patients, did not alter murine temperature sensitivity within the innocuous range but enhanced pain responses to noxious heat/cold[37]. In another study, the authors revealed that Parkinson mice (*Pink1*^*−/−*^*SNCA*^*A53T*^) exhibited analgesia in the Hotplate test but showed a preference for cooler temperatures when the Thermal Gradient Ring was utilized[38]. These paradoxical responses to heat sensation suggest that thermal preference within the innocuous temperature range and thermal avoidance/tolerance to pain induced by noxious temperature involve different activation mechanisms. This underscores the need for a multidimensional approach and methodology in interpreting such intricate behaviors. The Thermal Gradient Ring provides an alternative means to avoid arbitrary conclusions in the study of these complex phenomena.

A similar discrimination was applied in diabetic peripheral neuropathy (DPN) models. DPN includes symptoms of thermosensory impairment, which are reported to involve changes in the expression or function, or both, of nociceptive TRPV1 and TRPA1 channels in rodents [39–43]. A recent study showed that changes in the expression or function of TRPV1 or TRPA1 in DPN mice were caused by streptozotocin, whereas thermal hypoalgesia was observed in a murine model of DPN or in TRPV1^**−/−**^ mice using a plantar test [44], which specifically detects temperature avoidance. With a Thermal Gradient Ring, the temperature-dependent phenotype between DPN and TRPV1^**−/−**^ mice was clearly discriminated. Accordingly, approaches using multiple behavioral methods should be undertaken to analyze the progression of DPN and responses to thermal stimuli. Attention to both thermal avoidance and preference may provide more insight into DPN symptoms.

## Significance of TRPV3 in skin keratinocytes

Somatic thermosensation appears to involve a system that combines non-neuronal cells and sensory neurons. Keratinocytes are non-neuronal cells of ectoderm origin that reportedly contribute to somatic thermosensation. The skin is a large organ that represents the boundary between an organism and its environment, and consists almost entirely of keratinocytes. Keratinocytes respond to temperature stimulation via elevation in intracellular Ca^2+^. TRPV3 and TRPV4 are non-selective cation channels that are activated by warm temperatures and have high expression levels in skin keratinocytes [17, 20]. TRPV3 activation by warm temperatures was reported to evoke ATP release from keratinocytes and the released ATP subsequently activates ionotropic ATP receptors in peripheral nerves [45]. However, TRPV3/TRPV4 double KO mice showed normal behavioral responses to warm temperatures [46]. Therefore, the functions of TRPV3 and TRPV4 in somatic thermosensation are still not well-understood. Interestingly, optogenetic inhibition of keratinocytes impaired defensive responses to noxious cold and heat [47]. Nocifensive behaviors to noxious cold and heat were also blunted by intraplantar injection of apyrase, an enzyme that catalyzes ATP hydrolysis, as well as by sensory neuron-specific knockout of the P2X4 receptor [47]. These findings support a role for keratinocytes in somatic thermosensation that is mediated by purinergic signaling, even though the involvement of thermosensitive TRP channels is still unclear.

**Fig. 3:**
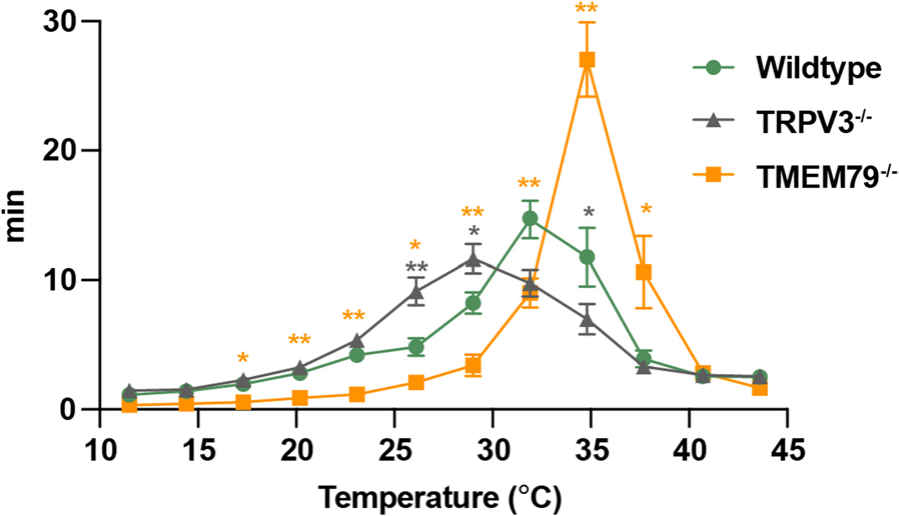
TMEM79-deficient mice exhibit a strong preference for a warmer temperature compared with wild-type mice. “Spent time” by wild-type, TRPV3^−/−^, or TMEM79^−/−^ mice in each temperature zone during a 1 h recording. The data were statistically analyzed with a mixed-effects ANOVA with Geisser-Greenhouse correction. All data and error bars represent the mean ± SEM. *, P < 0.05; **, P < 0.01. The Figure is adapted from ref. [48]

Lei et al. [48] provided evidence for the involvement of skin TRPV3 in temperature detection by showing that TRPV3 makes a complex with the membrane protein TMEM79 and formation of this complex is associated with reduced TRPV3 expression on the plasma membrane. Skin keratinocytes from TMEM79-deficient mice express high levels of TRPV3 proteins and showed increased TRPV3-mediated current responses. In contrast to WT mice that consistently showed a moderate preference for temperatures between 29 °C and 34.8 °C, TRPV3^−/−^ mice showed a wider range of preference (26.1 °C to 34.8 °C) that was consistent with previous studies [17, 32] (Fig. 3). In a Thermal Gradient Ring experiment, TMEM79-deficient mice preferred a higher temperature than did WT mice [48]. Notably, TRPV3^−/−^ mice and WT mice showed similar preferences at temperature zones below 23.1 °C and above 37.7 °C. By analyzing the exact peak temperature, WT mice were found to favor 30.4 ± 0.5 °C, whereas TRPV3^−/−^ mice had decreased peak temperature of 29.1 ± 0.4 °C that demonstrates involvement of TRPV3 in warm temperature perception. Interestingly, TMEM79^−/−^ mice had a narrower range of temperature preference compared to WT and TRPV3^−/−^ mice. TMEM79^−/−^ mice spent nearly half their time in a temperature zone around 35 °C with a peak at 34.4 °C ± 0.4 °C. In addition, both WT and TMEM79^−/−^ mice began discriminating their optimal temperature after 20 min in the Ring, whereas TRPV3^−/−^ mice remained indifferent to warm temperature through the first 40 min. These results suggest that the altered thermal selection of TMEM79^−/−^ mice might actually be due to increased TRPV3 expression. These findings provide direct support for the underlying involvement of TRPV3 in skin thermosensation.

## Conclusion

Thermal Gradient Ring assays revealed several interesting phenomena regarding temperature-dependent behaviors of mice. Future studies using this system would enhance understanding of the molecular basis of thermal behaviors in mice, which could be helpful for developing strategies.

to affect responses to different temperature conditions in humans.

## Data Availability

All data and materials used in the analysis are available in the manuscript or the cited works by the authors.
